# 

**Published:** 1897-12

**Authors:** 


### SOUTHERN DENTAL ASSOCIATION.

The next meeting of this body will be held at St. Augustine, Florida, in February, 1898.

Thus under the old name is this announcement made, and to all appearances it is the same in fact. To this no one can object, for it was an understood conclusion, at Old Point Comfort, that it was to live, practically the same body as heretofore. If the logic of the situation had been as well understood by the members of the American Dental Association they would not have permitted it to pass out of existence.

In the language of the President, in the circular issued, it is to be in the future "an organization now of Southern dentists, *by Southern dentists and for Southern dentists*. . . . We are proud (the circular continues) of the Southern's past; we will be more proud of it in the future. We have a chance to show what we can do. We of the South are not behind any people upon earth. Our history has been a glorious one. We are descended from the cream of 'Old England,' and are less contaminated to-day by undesirable foreign elements than any part of this great Union. . . . They have now an organization all their own. They try to rival no one; but propose to show to the world what they can do for dentistry."

Whether this grandiloquent message will serve to arouse enthusiasm or not, it certainly exhibits a self-satisfied condition of mind, and shows a determination to keep the Southern intact, and that upon a purely sectional basis. The outcome will be awaited with interest, for upon that will depend the solution of the question, now somewhat in doubt, Did the Southern really unite with the American Dental Association to form a new body?

---

### CORRECTION.

In the preparation of Dr. Davenport's article in the October number, an error occurred in the preparation for the press in reversing Figs. 1 and 2. They will, however, be readily understood as they stand upon the page.

### INDEX TO VOLUME XVIII.

A.

Abbott, Professor Frank, 345, 418, 479.
A Blot on the Profession, 199.
Abscess, Alveolar, A High Voltage Current for Treatment of, 155.
Abscess, Blind, 506.
Abstracts and Translations:
A Method of Strengthening Vulcanite Plates, 19.
Carbolic Acid as a Disinfectant, 511.
Formaldehyde, 444, 734.
Gutta-Percha, 651.
Investigations of the Nerves of the Pulp by the Methylene-Blue Method, 255.
Leptothrix Buccalis, A Few Considerations on, 816.
Silver and its Salts as Surgical Antiseptics, 101.
The Decay of the Teeth, 160.
The Discoloration of Copper Amalgam, 315.
The Microscopical Aspect of Certain Lesions induced by Dental Caries, 392.
The New Antiseptics of Dr. Credé: Silver and the Silver Salts, and their Use in Dentistry, 508.
Academy of Stomatology, 30, 175, 332, 526, 675.
Acetanilide as a Dressing, 701.
A Chapter in Dental History and Bibliography, 431.
A Contribution to the Study of the Development of Dental Enamel, 205.
A Dangerous Popular Antiseptic, 547.
Address, Annual, 90, 157 247.
A Description of some English Appliances, 497.
A Help for Insomnia, 624.
A High Voltage Current for a Threatened Alveolar Abscess, 155.
Allen, Dr. Harrison. Obituary, 844.
Aluminum Crowns, 352.
Alveolar Abscess, A High Voltage Current for, 155.
Alveolar Inflammations, Electricity in, 387.
Alveolaris, Radiography in Pyorrhœa, 140.
Amalgam, The Discoloration of Copper, 315.
American Academy of Dental Science, 321, 354, 449, 680.
American Academy of Dental Science, Remarks made at the Anniversary Dinner of, 502.
American and Southern Dental Associations, Union of, 236.
American Dental Association, 21, 105, 169, 482, 586, 657, 740, 822.
American Dental Association and Reorganization, 467.
American Dental Association, President's Address to the, 563.
A Method of Strengthening Vulcanite Plates, 19.
Anæsthetic, A Non-Toxic Local, 98.
Anatomy, Descriptive and Surgical. By Henry Gray, F.R.S. A Review of, 839.
Anatomy, The Study of, 581.
Ancient Metallurgy, 574.
A New Method of Treatment: Root-Perforation, 357.
Andrews, R. R., D.D.S. On A Contribution to the Study of the Development of Dental Enamel, 205.
Review of Dr. Black's Conclusions, 785.
An Incident in Dr. Garretson's Life, 480.
Anniversary Dinner of the American Academy of Dental Science in Boston, November 11, 1896, Remarks made at, 502.
Annual Address, 90, 157, 247.
Annual Dinner.—Honors to Dr. Benjamin Lord, 276.
Annual of the Universal Medical Sciences and Analytical Index, 346.
A Non-Toxic Local Anæsthetic, 98.
An Open Letter.—Examining Boards, 729.
Antagonism of Law, The, 614.
Antiseptic, A Dangerous Popular, 547.
Antiseptics, Silver and its Salts as Surgical, 101.
Antiseptic Treatment, A New Mode of: Formalin Gelatin, 203.
Antiseptic Treatment of Root-Canals, Some Points in the, 364.
Antrum, A Study of the Relation of the Frontal Sinus to the, 14.
Appliances, A Description of some English, 497.
A Practical Treatise on Artificial Crown- and Bridge-Work. By George Evans, D.D.S., 281.
A Practical Treatise on Mechanical Dentistry. By Joseph Richardson. Revised and edited by George W. Warren, D.D.S., 537.
Are We all at Sea? 197.
Arrington, B. F., Goldsboro, N. C. Examining Boards.—An Open Letter, 729.

Artificial Anæsthesia. By Lawrence Turnbull, M.D., Ph.G., 417.
Association, Southern Dental, 835.
Associations, Consolidation of the Two, 314.
A Study of the Relation of the Frontal Sinus to the Antrum, 14.
A Transplantation, The Technique of, 494.
Avery, Otis, D.D.S. Reminiscences of Sixty-four Years of Practice, 421.

B.

Ball, M.V., M.D. On The Mouth is the *Via Natura* for the Entrance of Disease, 724.
Balsamo del Platto, 352.
Barrett, W. C., M.D., D.D.S. The Study of Anatomy, 581.
Bethel, L. P., M.D., D.D.S. On The Use of Silver Salts in the Treatment of Root-Canals, 485.
BIBLIOGRAPHY:
- A Manual on the Injuries and Surgical Treatment of the Face, Mouth, and Jaws. By John S. Marshall, M.D., 836.
- Anatomy, Descriptive and Surgical. By Henry Gray, F.R.S., 839.
- Annual of the Universal Medical Sciences and Analytical Index. By Charles E. Sajous, M.D., Paris, 346.
- A Practical Treatise on Artificial Crown- and Bridge-Work. By George Evans, 281.
- A Practical Treatise on Mechanical Dentistry. By Joseph Richardson. Revised and edited by George W. Warren, D.D.S., 537.
- Artificial Anæsthesia. By Lawrence Turnbull, M.D., Ph.G., 417.
- Catching's Compendium of Practical Dentistry for 1896, 348.
- Chronic Alveolitis (Pyorrhœa Alveolairs). By Dr. Henry S. Nash, 772.
- Clinique Dentaire et Dentisterie Opératoire. By Ch. Godon, 348.
- Compend of Dental Pathology and Therapeutics. By Henry H. Burchard, M.D., D.D.S., 62.
- Dental Materia Medica, Pharmacology, and Therapeutics. By Charles W. Glassington, Edinburgh, Scotland, 192.
- *Items of Interest* for January, 1897, 192.
- Methods and Appliances in Operative and Mechanical Dentistry. By R. P. Lennox, 538.
- The American Text-Book of Operative Dentistry. In Contributions by Eminent Authorities. Edited by Edward C. Kirk, D.D.S., 616.
- Transactions of the American Dental Association at the Thirty-sixth Annual Session, 345.

Bibliography, A Chapter in Dental History and, 431.
Black's, Dr. G. V., Conclusions reviewed, 781, 785, 789, 796, 807, 812, 814.
Blakeney, Dr. Henry F., 351.
Bleaching, Methods of Tooth-, 198.
Blind Abscess, What is called a? 506.
Board of Registration in Dentistry, 355.
Board of Registration in Dentistry, Massachusetts, 701.
Bogue, E. A., D.D.S. Review of Dr. Black's Conclusions, 312.
Bridge-Work, Some Features in, 641.
Brigiotti, J. G. On Defective Articulation accompanied by Pain corrected by opening the Bite and the Insertion of Immovable Bridges, 439.
Brockway, Albert H., M.D.S. Cleansing Teeth, 507.
Brown, Dr. George C., Resolutions of Respect, 193.
Burchard, Henry H. Compend of Dental Pathology and Therapeutics, 62.
- On Hypersensitivity of Dentine and its Treatment, 1.
- The Work of James Edmund Garretson, 151.

C.

Calculus in Sublingual Gland, 63.
Carbolic Acid as a Disinfectant, 511.
Carbolic Acid, Lysol to replace, 547.
Care and Management of Deciduous Teeth, 85.
Caries, The Microscopical Aspect of Certain Lesions induced by Dental, 392.
Case, C. S., D.D.S., M.D. On Principles of Force and Anchorage in the Movement of the Teeth, 744.
Cassidy, J. S., D.D.S. On Relations of Chemistry to Dentistry, 719.
Cataphoresis, 549.
Cataphoresis, Experiments in, 64.
Cataphoresis, Methods of Controlling the Electric Current in, 297.
Cataphoresis, The Physiology of, 647.
Cataphoresis, The Value of Statistics in, 11.
Catching, Dr. B. H. On Consolidation of the Two Associations, 314.
Catching's Compendium of Practical Dentistry for 1896, 348.
Cells, Origin of Giant, 548.
Cements, Preparation of Dental Alloys and, 634.
Central Dental Association of Northern New Jersey, 43, 184, 202.
Cheever, David W. Annual Address, 157.
Chemistry, Relations of, to Dentistry, 719.
Cheney, Charles D. On Nitrate of Silver in Root-Canals, 99.
Choquet, T., D.E.D.P. A Few Considerations on the Leptothrix Buccalis, 816.
Chronic Alveolitis (Pyorrhœa Alveolaris), 772.
Cleansing Teeth, 507.
Clinical Report on Method of Operating, 636.
Clinique Dentaire et Dentisterie Opératoire, By Ch. Godon, 348.

Coagulation Theory, The, 536.
Cocaine, 702.
Communications on Theory and Practice, 121, 402, 458.
Compend of Dental Pathology and Therapeutics. By Henry H. Burchard, M.D., D.D.S., 62.
Consolidation of the Two Associations, 314.
Cope, Edward Drinker, 344, 350.
Copper Amalgam, The Discoloration of, 315.
Correction, 137, 197, 482, 835.
Credé, Dr. B. On Silver and its Salts as Surgical Antiseptics, 101.
"Crime, Not Failure, but Low Aim is," 310.
Criticisms, Reply to, 415.
Crowns, Aluminum, 352.
Crown- and Bridge-work, 285.
Crown- and Bridge-Work, A Practical Treatise in Artificial, 281.
Crown- and Bridge-Work, Removable Porcelain, 306.
Crowns, Gold-Shell, One Hundred and Fifty Years Ago, 639.
Current Literature, Report on, 513.
CURRENT NEWS:
- A Voluntary Statement by Dr. Joseph Head, which was accepted by the Pennsylvania State Dental Society, July, 1897, 542.
- Board of Registration in Dentistry, 355.
- Board of Registration in Dentistry, Massachusetts, 701.
- Central Dental Association of Northern New Jersey, 184, 202.
- Dental Society of the State of New York, 200, 282.
- Harvard Odontological Society, 283, 530.
- Meeting of the American Medical Association, 353.
- Michigan Dental Association, 355.
- National Association of Dental Examiners, 483, 700.
- National Association of Dental Faculties, 482, 605.
- National Association of Dental Faculties, Report of the Committee on Necrology of the, 775.
- New England Association of Dental Examiners, 484.
- New Jersey State Dental Society, 483, 624.
- New York Institute of Stomatology, 68.
- Northern Illinois Dental Society, 847.
- Northern Ohio Dental Society, 546.
- Odontographic Society of Chicago, 202.
- Pennsylvania Association of Dental Surgeons, 68.
- Pennsylvania State Dental Examining Board, 420, 701.
- Pennsylvania State Dental Society, 420.
- Postponement of Meeting, 546.

CURRENT NEWS:
- Programme of the Rochester Dental Society, 846.
- Report of the Committee on Irregularity at the Pennsylvania State Dental Society, Glen Summit, Pa., July 6, 1897, 541.
- Resolutions of American Academy of Dental Science, 354.
- Resolutions of the Odontological Society of Pennsylvania, 420.
- St. Louis Dental Society, Officers for 1897, 202.
- The Harvard Dental Alumni Association, 543.
- The International Tooth-Crown Company *versus* Allan G. Bennett, 65.
- Twelfth International Medical Congress, 200.
- Woman's Dental Association of the United States, 544.

Custer, L. E., B.S., D.D.S. The Value of Statistics in Cataphoresis, 11.

D.

Davenport, S. E. On the Restoration of Badly Broken Teeth without Crowning, 240.
Davenport, Wm. Slocum, D.D.S. On Regulating without and with Extraction, 625, 777.
Deciduous Teeth, Some Thoughts on the Care and Management of, 85.
Defective Articulation accompanied by Pain corrected by Opening the Bite and the Insertion of Immovable Bridges, 439.
Degenerate Jaws and Teeth, The, 69, 141, 225.
Dental Alloys, Preparation of, 634.
Dental Colleges, Entrance Examinations in, 697.
Dental Caries, Relation of Tuberculous Glands in the Neck to, 351.
Dental Caries, The Microscopical Aspect of Certain Lesions Induced by, 392.
Dental Enamel, A Contribution to the Study of the Development of, 205.
Dental Examiners, New England Association of, 484.
Dental Examiners, National Association of, 483, 700.
Dental Examining Board, Pennsylvania State, 420.
Dental Faculties, National Association of, 482, 605, 775.
Dental Gold-Mining Company, International, 624.
Dental History and Bibliography, A Chapter in, 431.
Dental Materia Medica, Pharmacology, and Therapeutics. By Charles W. Glassington, Edinburgh, Scotland, 192.
Dental Nostrums, 534.
Dental Society of the State of New York, 200, 282.
Dentine, Hypersensitivity of, 1.

Dentistry, Electricity and, 631.
Dentistry, Relations of Chemistry to, 719.
Devitalized Teeth, Treatment of, 370.
Diphtheria, 705.
Discoloration of Copper Amalgam, 315.
Disease, The Mouth is the *Via Natura* for the Entrance of, 724.
Disinfectant, Carbolic Acid as a, 511.
DOMESTIC CORRESPONDENCE:
An Incident in Dr. Garretson's Life, 480.
Calculus in Sublingual Gland, 63.
Correction, 197, 482.
Experiments in Cataphoresis, 64.
Radiography in Pyorrhœa Alveolaris, 140.
Reply of Dr. C. N. Peirce, 540.
Reply of Dr. Faught, 700.
Reply to Dr. C. N. Peirce, 481.
Dr. Bonwill's Visit to Europe, 343.
Dr. Farrar's Second Volume, 416.
Dr. Francis Peabody, 194.
Dr. James A. Swasey, 195.
Dr. Robinson *versus* Dr. Younger, in Reference to Implantation, 388.

E.

EDITORIALS:
American Dental Association and Reorganization, 467.
Dental Nostrums, 534.
Dr. Bonwill's Visit to Europe, 343.
Dr. Farrar's Second Volume, 416.
Editors not always Responsible, 472.
Edward Drinker Cope, 344.
Eighty-nine and in Practice, 474.
Is it Just? 280.
John C. Storey, M.D., D.D.S., 343.
Old Point Comfort, 611.
Pennsylvania State Dental Society, 535.
Principles governing Malleting Force, 133.
Professor Frank Abbott, 345.
Reorganization, 278.
Reply to Criticisms, 415.
Southern Dental Association 835.
The American Medical Association, 471.
The Antagonism of Law, 614.
The Coagulation Theory, 536.
The Death of Dr. Emile Magitot, 475.
The Entrance Examinations in Dental Colleges, 697.
The Evolution and Abuse of the Serration, 188.
Then and Now, 338.
The Needs of the Future, 58.
The Record of the Year, 832.
The Reign of Law, 768.
Editors not always Responsible, 472.
Eighty-nine and in Practice, 474.
Election of Officers, 173.
Electrical Irritation of Fillings, 156.
Electric Current, Methods of controlling, in Cataphoresis, 297.
Electricity and Dentistry, 631.
Electricity in Alveolar Inflammations, 387.
Elliott, William St. George, Jr. On Methods of controlling the Electric Current in Cataphoresis, 297.
Enamel, A Contribution to the Study of the Development of Dental, 205.
English Appliances, A Description of some, 497.
Entrance of Disease, The Mouth is the *Via Natura* for the, 724.
Evans, George. A Practical Treatise on Artificial Crown- and Bridge-Work, 281.
Evans, Dr. Thomas W. Obituary, 841.
Examining Boards.—An Open Letter, 729.
Experiments in Cataphoresis, 64.
Extraction, Regulating without and with, 625, 777.

F.

Fillebrown, Dr. Thomas. A Study of the Relation of the Frontal Sinus to the Antrum, 14.
On Union of the American and Southern Dental Associations, 236.
Fillings, Electrical Irritation of, 156.
Five-Minute Papers, 96.
Flickinger, Adam, D.D.S. On Removable Porcelain Crown- and Bridge-Work, 306.
Formaldehyde, 444, 734.
Formalin Gelatin: A New Mode of Antiseptic Treatment, 203.
Frontal Sinus, Relation of the, to the Antrum, 14.
Future, The Needs of the, 58.

G.

Garretson's Life, An Incident in Dr., 480.
Garretson, The Work of James Edmund, 151.
Gethins, James L. On Electricity and Dentistry, 631.
Giant Cells, Origin of, 548.
Gilliams, J. S., M.D., D.D.S. On Some Dental Manifestations of Gout, 441.
Girdwood, John, D.D.S., L.D.S., Edinburgh, Scotland. On Root-Perforation: A New Method of Treatment, 357.
Gland, Calculus in Sublingual, 63.
Glassington, Charles W., L.D.S., Edinburgh, Scotland. Dental Materia Medica, Pharmacology, and Therapeutics, 192.
Goadby, Kenneth W. On The Discoloration of Copper Amalgam, 315.
Godon, Ch. Clinique Dentaire et Dentisterie Opératoire, 348.
Gold-Mining Company, International Dental, 624.
Gold-Shell Crowns One Hundred and Fifty Years Ago, 639.
Goldsmith, S. L. On Calculus in Sublingual Gland, 63.
Gout, Some Dental Manifestations of, 441.
Grant, George T., D.M.D. On a Description of some English Appliances, 497.
Gray, Henry, F.R.S. Anatomy, Revision of, 839.

Guilford, S. H. On Some Thoughts on the Care and Management of the Diciduous Teeth, 85.
Gutta-Percha, 651.

H.

Harvard Odontological Society, 283, 530.
Hayes, Dr. Samuel J., 623.
Hildreth, J. L., M.D. On To What Extent is Typhoid Fever Preventable? 373.
Hille, M., D.D.S. The New Antiseptics of Dr. Credé: Silver and the Silver Salts, and their Use in Dentistry, 508.
Hollænder, Professor Dr. Med. Ludw., 480.
Howe, J. Morgan, M.D., M.D.S. On Some Points in the Antiseptic Treatment of Root-Canals, 364.
Hypersensitivity of Dentine and its Treatment, 1.

I.

Immovable Bridges, Defective Articulation accompanied by Pain corrected by opening the Bite and the Insertion of, 439.
Implantation, Dr. Robinson *versus* Dr. Younger in Reference to, 388.
Inflammations, Electricity in Alveolar, 387.
Insomnia, A Help for, 624.
Institute of Stomatology, New York, 40, 68, 119, 263, 401, 457, 513, 588, 660, 826.
International Dental Gold-Mining Company, 624.
Investigations of the Nerves of the Pulp by the Methylene-Blue Method, 255.
Irritation of Fillings, Electrical, 156.
Is it Just? 280.
*Items of Interest* for January, 1897, 192.

[illegible]

Jack, Louis, D.D.S. On Cataphoresis, 549.
  On Dr. Robinson *versus* Dr. Younger in Reference to Implantation, 388.
  On The Technique of a Transplantation, 494.
  On Treatment of Devitalized Teeth, 370.
Jaws and Teeth, The Degenerate, 69, 141, 225.
Jones, W. H., D.D.S. On a Non-Toxic Local Anæsthetic, 98.
Jung, Professor. On the Decay of the Teeth, 160.
Just, Is it? 280.

K.

Kirk, Edward C., D.D.S. The American Text-Book of Operative Dentistry. In Contributions by Eminent Authorities, 616.

L.

Law, The Antagonism of, 614.
Law, The Reign of, 768.
Leffmann, Henry, M.D., D.D.S. On Ancient Metallurgy, 574.
Lennox, R. P. Some Methods and Appliances in Operative and Mechanical Dentistry, 538.
Leptothrix Buccalis, A Few Considerations on, 816.
Lesions induced by Dental Caries, The Microscopical Aspect of Certain, 392.
Lewis, J. Hale, D.D.S. Experiments in Cataphoresis, 64.
Literature, Report on Current, 513.
Local Anæsthetic, A Non-Toxic, 98.
Lord, Dr. Benjamin, Honors to, 276.
"Low Aim is Crime, Not Failure, but," 310.
Lysol to replace Carbolic Acid, 547.

M.

Magitot, Le Docteur Emile, 475, 476.
  Resolutions of Respect to, 776.
Malleting Force, Principles governing, 133.
Manifestations of Gout, Some Dental, 441.
Marshall, John S., M.D. A Manual on Injuries and Diseases of the Face, Mouth, and Jaws, A Review of, 836.
Massachusetts Dental Society, 694.
Maxfield, George A., D.D.S. Annual Address, 247.
  Review of Dr. Black's Conclusions, 807.
Medical Association, The American, 471.
Meeting of the American Medical Association, 353.
Metallurgy, Ancient, 574.
Method of Operating, Clinical Report of, 636.
Methods of Controlling the Electric Current in Cataphoresis, 297.
Methods of Tooth-Bleaching, 198.
Methylene-Blue Method, Investigations of the Nerves of the Pulp by the, 255.
Michigan Dental Association, 355.
Mills, Dr. G. A. On New Field suggested by Dr. Williams's Paper, 390.
Mills, Dr. S. Alden. What is called a Blind Abscess? 506.
McManus, Charles, D.D.S. On Five-Minute Papers, 96.
McManus, James, D.D.S. Annual Address, 90.
Morgenstern, Michael, Germany. On Investigations of the Nerves of the Pulp by Methylene-Blue Method, 255.
Morley, C. R., L.D.S. A Method of Strengthening Vulcanite Plates, 19.
Morrison, William Newton, D.D.S., 137.
Movement of the Teeth, Principles of Force and Anchorage in the, 744.

N.

National Association of Dental Examiners, 483, 700.
National Association of Dental Faculties, 482, 605.
National Association of Dental Faculties, Report of the Committee on Necrology of the, 775.

Needs of the Future, The, 58.
New England Association of Dental Examiners, 484.
New Field suggested by Dr. Williams's Paper, 390.
New Jersey State Dental Society, 483, 624.
New York Institute of Stomatology, 40, 68, 119, 263, 401, 457, 513, 588, 660, 826.
Nitrate of Silver in Root-Canals, 99.
Non-Toxic Local Anæsthetic, 98.
NOTES AND COMMENTS:
  A Blot on the Profession, 199.
  A Help for Insomnia, 624.
  Aluminum Crowns, 352.
  Are We all at Sea? 197.
  Balsamo del Platto, 352.
  International Dental Gold-Mining Company, 624.
  Methods of Tooth-Bleaching, 198.
  "Platir," 198.
  Relation of Tuberculous Glands in the Neck to Dental Caries, 351.
  The Benefit of Sunlight, 352.
"Not Failure, but Low Aim is Crime," 310.
Northern Illinois Dental Society, 847.
Northern Ohio Dental Society, 546.
Nostrums, Dental, 534.
Now, Then and, 338.

O.

OBITUARY:
  Abbott, Frank, M.D., 418, 479, 775.
  Allen, Dr. Harrison, 844.
  Blakeney, Dr. Henry F., 351.
  Cope, Professor Edward Drinker, 350.
  Evans, Dr. Thomas W., 841.
  Hayes, Dr. Samuel J., 623.
  Hollænder, Professor, 480.
  Magitot, Le Docteur, 475, 476.
  Morrison, William Newton, D.D.S., 137.
  Peabody, Dr. Francis, 194, 775.
  Report of the Committee on Necrology of the National Association of Dental Faculties, 775.
  Resolutions of Respect to Dr. E. Magitot, 776.
  Resolutions of Respect to Dr. George C. Brown, 193.
  Smith, Dr. Wm. Henry, 540.
  Storey, John C., M.D., D.D.S., 349.
  Swasey, Dr. James A., 195.
Odontographic Society of Chicago, 202.
Odontological Society of Pennsylvania, 598, 761.
Odontological Society of Pennsylvania, Resolutions of, 420.
Old Point Comfort, 611.
Operating, Clinical Report on Method of, 636.
ORIGINAL COMMUNICATIONS:
  A Chapter in Dental History and Bibliography, 431.
  A Contribution to the Study of the Development of Dental Enamel, 205.
  A Description of some English Appliances, 497.

ORIGINAL COMMUNICATIONS:
  A High Voltage Current will stop a Threatened Alveolar Abscess, 155.
  Ancient Metallurgy, 574.
  Annual Address, 90, 157, 247.
  A Non-Toxic Local Anæsthetic, 98.
  A Study of the Relation of the Frontal Sinus to the Antrum, 14.
  Cataphoresis, 549.
  Cleansing Teeth, 507.
  Clinical Report on Method of Operating, 636.
  Consolidation of the Two Associations, 314.
  Crown- and Bridge-Work, 285.
  Defective Articulation accompanied by Pain corrected by opening the Bite and the Insertion of Immovable Bridges, 439.
  Diphtheria, 705.
  Dr. Robinson *versus* Dr. Younger, in Reference to Implantation, 388.
  Electrical Irritation of Fillings, 156.
  Electricity and Dentistry, 631.
  Electricity in Alveolar Inflammations, 387.
  Examining Boards.—An Open Letter, 729.
  Gold-Shell Crowns One Hundred and Fifty Years Ago, 639.
  Hypersensitivity of Dentine and its Treatment, 1.
  Methods of Controlling the Electric Current in Cataphoresis, 297.
  New Field suggested by Dr. Williams's Paper, 390.
  Nitrate of Silver in Root-Canals, 99.
  "Not Failure, but Low Aim is Crime," 310.
  On Five-Minute Papers, 96.
  Preparation of Dental Alloys and Cements, 634.
  President's Address to the American Dental Association, 1897, 563.
  Principles of Force and Anchorage in the Movement of the Teeth, 744.
  Regulating without and with Extraction, 625.
  Relations of Chemistry to Dentistry, 719.
  Remarks made at Anniversary Dinner of the American Academy of Dental Science in Boston, November 11, 1896, 502.
  Reminiscences of Sixty-four Years of Practice, 421.
  Removable Porcelain Crown- and Bridge-Work, 306.
  Root-Perforation: A New Method of Treatment, 357.
  Some Dental Manifestations of Gout, 441.
  Some Features in Bridge-Work, 641.
  Some Points in the Antiseptic Treatment of Root-Canals, 364.
  Some Thoughts on the Care and Management of the Deciduous Teeth, 85.

ORIGINAL COMMUNICATIONS:
The Degenerate Jaws and Teeth, 69, 141, 225.
The Mouth is the *Via Natura* for the Entrance of Disease, 724.
The Physiology of Cataphoresis, 647.
The Restoration of Badly Broken Teeth without Crowning, 240.
The Study of Anatomy, 581.
The Technique of a Transplantation, 494.
The Use of Silver Salts in the Treatment of Root-Canals, 485.
The Value of Statistics in Cataphoresis, 11.
The Work of James Edmund Garretson, 151.
Third Set of Teeth, 499.
Thoughts on Regulating, 435.
To what Extent is Typhoid Fever Preventable? 373.
Treatment of Devitalized Teeth, 370.
Union of the American and Southern Dental Associations, 236.
What is called a Blind Abscess? 506.
Origin of Giant Cells, 548.
Oxidation, 203.

P.

Palmer, S. B., M.D.S. On Third Set of Teeth, 499.
Review of Dr. Black's Conclusions, 789.
Papers, Five-Minute, 96.
Peabody, Dr. Francis, 194.
Peeso, Fred. A., D.D.S. On Crown- and Bridge-Work, 285.
Peirce, Dr. C. N., Reply of, 540.
Peirce, Dr. C. N., Reply to, 481.
Pennsylvania Association of Dental Surgeons, 68.
Pennsylvania State Dental Examining Board, 420, 701.
Pennsylvania State Dental Society, 420, 535.
Pennsylvania State Dental Society at Glen Summit, Pa., July 6, 1897, 541.
Pennsylvania State Dental Society, Report of Committee on Irregularity at the, 541.
Pennsylvania State Dental Society, Voluntary Statement by Dr. Joseph Head, which was accepted by, 542.
Perforation, Root-: A New Method of Treatment, 357.
Peroxide of Hydrogen in Wound Treatment, 284.
Physiology of Cataphoresis, The, 647.
"Platir," 198.
Popular Antiseptic, A Dangerous, 547.
Porter, Dr. A. H. On Electrical Irritation of Fillings, 156.
Postponement of Meeting, 546.
Practice, Communications on Theory and, 121, 402, 458.
Practice, Eighty-nine and in, 474.
Practice, Reminiscences of Sixty-four Years of, 421.
Preparation of Dental Alloys and Cements, 634.
President's Address to the American Dental Association, 1897, 563.
Price, Weston A. On the Physiology of Cataphoresis, 647.
Principles governing Malleting Force, 133.
Principles of Force and Anchorage in the Movement of the Teeth, 744.
Profession, A Blot on the, 199.
Pulp, Investigations of the Nerves of the, by the Methylene-Blue Method, 255.
Pyorrhœa Alveolaris (Chronic Alveolitis), 772.
Pyorrhœa Alveolaris, Radiography in, 140.

R.

Radiography in Pyorrhœa Alveolaris, 140.
Recent Patents, 283, 356, 545.
Register, Dr. H. C. On Clinical Report on Method of Operating, 636.
Some Features in Bridge-Work, 641.
Regulating, Thoughts on, 435.
Regulating without and with Extraction, 625, 777.
Reign of Law, The, 768.
Relation of the Frontal Sinus to the Antrum, A Study of the, 14.
Relation of Tuberculous Glands in the Neck to Dental Caries, 351.
Relations of Chemistry to Dentistry, 719.
Remarks made at Anniversary Dinner of the American Academy of Dental Science in Boston, November 11, 1896, 502.
Reminiscences of Sixty-four Years of Practice, 421.
Removable Porcelain Crown- and Bridge-Work, 306.
Reorganization, 278.
Reorganization, American Dental Association and, 467.
Reply of Dr. C. N. Peirce, 540.
Reply of Dr. Faught, 700.
Reply to Criticisms, 415.
Reply to Dr. C. N. Peirce, 481.
REPORTS OF SOCIETY MEETINGS:
Academy of Stomatology, 30, 175, 332, 526, 675.
American Academy of Dental Science, 321, 354, 449, 680.
American Dental Association, 21, 105, 169, 482, 586, 657, 740, 822.
Central Dental Association of Northern New Jersy, 43, 184, 202.
Massachusetts Dental Society, 694.
New York Institute of Stomatology, 40, 68, 119, 263, 401, 457, 513, 588, 660, 826.
Odontological Society of Pennsylvania, 598, 761.
Report of the Committee on Irregularity at the Pennsylvania State Dental Society at Glen Summit, Pa., July 6, 1897, 541.
Report of the Committee on Necrology of the National Association of Dental Faculties, 775.

Report on Current Literature, 518.
Resolutions of American Academy of Dental Science, 354.
Resolutions of Respect to Dr. George C. Brown, 193.
Resolutions of Respect to Dr. E. Magitot, 776.
Resolutions of the Odontological Society of Pennsylvania, 420.
Responsible, Editors not always, 472.
Restoration of Badly Broken Teeth without Crowning, 240.
Richardson's Mechanical Dentistry. Edited by George W. Warren, D.D.S. A Review of, 537.
Roberts, Howard E., D.D.S. Thoughts on Regulating, 435.
Rochester Dental Society, Programme of, 1897–98, 846.
Rollins, William. On A High Voltage Current will stop a Threatened Alveolar Abscess, 155.
  On Electricity in Alveolar Inflammations, 387.
  On Radiography in Pyorrhœa Alveolaris, 140.
Root-Canals, Nitrate of Silver in, 99.
Root-Canals, Silver Salts in the Treatment of, 485.
Root-Canals, Some Points in the Antiseptic Treatment of, 364.
Root-Perforation: A New Method of Treatment, 357.

S.

Sajous, Charles E., M.D. Annual of the Universal Medical Sciences and Analytical Index, 346.
Selections:
  Acetanilide as a Dressing, 701.
  A Dangerous Popular Antiseptic, 547.
  Carious Teeth as a Means of Entry for the Bacillus of Tuberculosis, 848.
  Cocaine, 702.
  Formalin Gelatin: A New Mode of Antiseptic Treatment, 203.
  Lysol to replace Carbolic Acid, 547.
  Origin of Giant Cells, 548.
  Oxidation, 203.
  Peroxide of Hydrogen in Wound Treatment, 284.
  Urotropine, 703.
Serration, The Evolution and Abuse of the, 188.
Shaw, Louis, M.D. Preparation of Dental Alloys and Cements, 634.
Silver and its Salts as Surgical Antiseptics, 101.
Silver and the Silver Salts: The New Antiseptics of Dr. Credé, and their Use in Dentistry, 508.
Silver, Nitrate of, in Root-Canals, 99.
Silver Salts, The Use of, in the Treatment of Root-Canals, 485.
Sixty-four Years of Practice, Reminiscences of, 421.
Smith, A. Hopewell, L.D.S. On the Microscopical Aspect of Certain Lesions induced by Dental Caries, 392.
Smith, B. Holly, D.D.S. Review of Dr. Black's Conclusions, 814.
Smith, Dr. William Henry, 540.
Some Dental Manifestations of Gout, 441.
Some English Appliances, A Description of, 497.
Some Features in Bridge-Work, 641.
Some Methods and Appliances in Operative and Mechanical Dentistry. By R. P. Lennox, 538.
Some Points in the Antiseptic Treatment of Root-Canals, 364.
Some Thoughts on the Care and Management of the Deciduous Teeth, 85.
Southern Dental Association, Union of the American and, 236, 835.
Statistics in Cataphoresis, The Value of, 11.
Strengthening Vulcanite Plates, A Method of, 19.
St. Louis Dental Society.—Officers for 1897, 202.
Stomatology, Academy of, 30, 175, 332, 526, 675.
Stomatology, New York Institute of, 40, 68, 119, 263, 401, 457, 513, 588, 660, 826.
Storey, John C., M.D., D.D.S., 343, 349.
Study of the Development of Dental Enamel, A Contribution to the, 205.
Sublingual Gland, Calculus in, 63.
Sunlight, The Benefit of, 352.
Surgical Antiseptics, Silver and its Salts as, 101.
Swarts, Gardner T., M.D. On Diphtheria, 705.
Swasey, Dr. James A., 195.

T.

Talbot, Eugene S., M.D., D.D.S. On the Degenerate Jaws and Teeth, 69, 141, 225.
Technique of a Transplantation, The, 494.
Teeth, Cleansing, 507.
Teeth, Restoration of Badly Broken, 240.
Teeth, Some Thoughts on the Care and Management of Deciduous, 85.
Teeth, The Decay of the, 160.
Teeth, The Degenerate Jaws and, 69, 141, 225.
Teeth, Third Set of, 499.
Teeth, Treatment of Devitalized, 370.
The American Medical Association, 471.
The American Text-Book of Operative Dentistry. In Contributions by Eminent Authorities. Edited by Edward C. Kirk, D.D.S., 616.
The Antagonism of Law, 614.
The Benefit of Sunlight, 352.
The Coagulation Theory, 536.
The Death of Dr. Emile Magitot, 475.
The Decay of the Teeth, 160.
The Degenerate Jaws and Teeth, 69, 141, 225.
The Discoloration of Copper Amalgam, 315.

The Entrance Examinations in Dental Colleges, 697.
The Evolution and Abuse of the Serration, 188.
The Harvard Dental Alumni Association, 543.
The International Tooth-Crown Company *versus* Allan G. Bennett, 65.
The Microscopical Aspect of Certain Lesions induced by Dental Caries, 392.
The Mouth is the *Via Natura* for the Entrance of Disease, 724.
The Needs of the Future, 58.
The New Antiseptics of Dr. Credé: Silver and the Silver Salts, and their Use in Dentistry, 508.
The Physiology of Cataphoresis, 647.
The Record of the Year, 832.
The Reign of Law, 768.
The Restoration of Badly Broken Teeth without Crowning, 240.
The Study of Anatomy, 581.
The Technique of a Transplantation, 494.
The Use of Silver Salts in the Treatment of Root-Canals, 485.
The Value of Statistics in Cataphoresis, 11.
The Work of James Edmund Garretson, 151.
Then and Now, 338.
Theory and Practice, Communications on, 121, 402, 458.
Theory, The Coagulation, 536.
Third Set of Teeth, 499.
Thoughts on Regulating, 435.
Tomes, Charles S., F.R.S. Review of Dr. Black's Conclusions, 781.
Tooth-Bleaching, Methods of, 198.
To what Extent is Typhoid Fever Preventable? 373.
Transactions of the American Dental Association at the Thirty-sixth Annual Session, 345.
Transplantation, The Technique of a, 494.
Treatment, A New Method of, 357.
Treatment of Devitalized Teeth, 370.
Treatment of Hypersensitivity of Dentine, 1.
Treatment of Root-Canals, Silver Salts in the, 485.
Treatment of Root-Canals, Some Points in the Antiseptic, 364.
Treatment, Peroxide of Hydrogen in Wound, 284.
Treatment, Root-Perforation: A New Method of, 357.
Trueman, William H., D.D.S. On A Chapter in Dental History and Bibliography, 431.
    Gold-Shell Crowns One Hundred and Fifty Years Ago, 639.
Truman, James, D.D.S. President's Address to the American Dental Association, 1897, 563.
    Review of Dr. Black's Conclusions, 796.
Tuberculous Glands in the Neck, Relation of, to Dental Caries, 351.
Turnbull, Lawrence, M.D., Ph.G. On Artificial Anæsthesia, 417.
Twelfth International Medical Congress, 200.
Typhoid Fever, To what Extent is it Preventable? 373.

U.

Union of the American and Southern Dental Associations, 236.
Urotropine, 703.

V.

Vulcanite Plates, A Method of Strengthening, 19.

W.

Warren, George W., D.D.S. Revision of Richardson's Mechanical Dentistry, 537.
Werner, J. G. W., D.M.D. On "Not Failure, but Low Aim is Crime," 310.
What is called a Blind Abscess? 506.
Williams, Jacob L., M.D. Remarks made at Anniversary Dinner of the American Academy of Dental Science in Boston, November 11, 1896, 502.
Williams's Paper, New Field suggested by Dr., 890.
Woman's Dental Association of the United States, 544.
Wood, H. C., M.D., LL.D. On Formaldehyde, 734.
Wound Treatment, Peroxide of Hydrogen in, 284.

Z.

Zahn und Mundleiden mit Bezug auf Allgemein-Erkrankungen. Ein Wegweiser für Aerzte und Zahnärzte. Von Zahnarzt P. Ritter, 840.

